# Epidemiology of respiratory viral infections in two long-term refugee camps in Kenya, 2007-2010

**DOI:** 10.1186/1471-2334-12-7

**Published:** 2012-01-17

**Authors:** Jamal A Ahmed, Mark A Katz, Eric Auko, M Kariuki Njenga, Michelle Weinberg, Bryan K Kapella, Heather Burke, Raymond Nyoka, Anthony Gichangi, Lilian W Waiboci, Abdirahman Mahamud, Mohamed Qassim, Babu Swai, Burton Wagacha, David Mutonga, Margaret Nguhi, Robert F Breiman, Rachel B Eidex

**Affiliations:** 1 US Centers for Disease Control and Prevention, Nairobi, Kenya; 2 Kenya Medical Research Institute, Nairobi, Kenya; 3 US Centers for Disease Control and Prevention, Atlanta, GA, USA; 4 United Nations High Commissioner for Refugees, Nairobi, Kenya; 5 Ministry of Public Health and Sanitation, Nairobi, Kenya; 6 International Rescue Committee, Nairobi, Kenya; 7 KEMRI/CDC, Mbagathi Road off Mbagathi Way, KEMRI Compound, P.O. Box 606, Nairobi 00621, Kenya

## Abstract

**Background:**

Refugees are at risk for poor outcomes from acute respiratory infections (ARI) because of overcrowding, suboptimal living conditions, and malnutrition. We implemented surveillance for respiratory viruses in Dadaab and Kakuma refugee camps in Kenya to characterize their role in the epidemiology of ARI among refugees.

**Methods:**

From 1 September 2007 through 31 August 2010, we obtained nasopharyngeal (NP) and oropharyngeal (OP) specimens from patients with influenza-like illness (ILI) or severe acute respiratory infections (SARI) and tested them by RT-PCR for adenovirus (AdV), respiratory syncytial virus (RSV), human metapneumovirus (hMPV), parainfluenza viruses (PIV), and influenza A and B viruses. Definitions for ILI and SARI were adapted from those of the World Health Organization. Proportions of cases associated with viral aetiology were calculated by camp and by clinical case definition. In addition, for children < 5 years only, crude estimates of rates due to SARI per 1000 were obtained.

**Results:**

We tested specimens from 1815 ILI and 4449 SARI patients (median age = 1 year). Proportion positive for virus were AdV, 21.7%; RSV, 12.5%; hMPV, 5.7%; PIV, 9.4%; influenza A, 9.7%; and influenza B, 2.6%; 49.8% were positive for at least one virus. The annual rate of SARI hospitalisation for 2007-2010 was 57 per 1000 children per year. Virus-positive hospitalisation rates were 14 for AdV; 9 for RSV; 6 for PIV; 4 for hMPV; 5 for influenza A; and 1 for influenza B. The rate of SARI hospitalisation was highest in children < 1 year old (156 per 1000 child-years). The ratio of rates for children < 1 year and 1 to < 5 years old was 3.7:1 for AdV, 5.5:1 for RSV, 4.4:1 for PIV, 5.1:1 for hMPV, 3.2:1 for influenza A, and 2.2:1 for influenza B. While SARI hospitalisation rates peaked from November to February in Dadaab, no distinct seasonality was observed in Kakuma.

**Conclusions:**

Respiratory viral infections, particularly RSV and AdV, were associated with high rates of illness and make up a substantial portion of respiratory infection in these two refugee settings.

## Background

The World Health Organization (WHO) estimates that acute lower respiratory infections (ALRI) cause nearly four million deaths per year, a rate of more than 60 deaths/100,000 population [1]. Rates are even higher in developing countries, where pneumonia is responsible for an estimated 10-25% of all deaths among children under 5 years of age [2]. While bacterial infections, especially due to *Streptococcus pneumoniae*, are critically important, viral causes are also associated with a substantial proportion of ALRI [3-7]. Viral infections can exacerbate chronic or recurring respiratory conditions, including asthma, thus representing an additional burden of respiratory viruses [8,9].

Respiratory syncytial virus (RSV) and influenza viruses are important pathogens in childhood pneumonia, and primary infection with these two pathogens predisposes children to secondary bacterial pneumonia, especially in children < 2 years old [4,6,7,10,11]. RSV has been identified in 15-40% of cases in hospitalised children, while human metapneumovirus (hMPV) and parainfluenza viruses (PIV) are associated with a substantial proportion of ALRI in infants and young children; asymptomatic infection with these pathogens appears uncommon [4,6,7,12-14]. The United Nations High Commissioner for Refugees (UNHCR) reports ALRI as the leading cause of mortality and morbidity among refugees in Kenya, where 30-40% of deaths of children < 5 years of age and up to 45% of morbidity is associated with acute respiratory infections [1,15]. For viral respiratory pathogens, vaccines are currently available only to prevent influenza; however, vaccine research and development are focusing on many other leading viral pathogens [10,16-18]. Understanding the epidemiology of specific aetiologies of ALRI in Africa is therefore important if maximal benefit is to be obtained from new vaccine developments [19].

Displaced populations, including refugees, are at risk for negative outcomes secondary to ALRI because of malnutrition, poor living conditions, and overcrowding [12,20-22]. At the end of 2009, there were 15.2 million refugees in the world, and some 5.2 million were in protracted situations [23]. Due to chronic intractable conflicts in some parts of the world, many refugees live in long-term camps for years or decades, with unique challenges that are often as difficult as the crisis management necessary during the emergency phase of refugee relief operations [24]. In some settings, additional challenges are posed by sustained or intermittent bursts of large numbers of new refugee arrivals from areas of renewed conflict [23].

In 2006, as the concern for human pandemic influenza grew, the Kenya Ministry of Health, in collaboration with the United States Centers for Disease Control and Prevention (CDC), established a nationwide facility-based influenza sentinel surveillance network comprising 11 sites, including Kakuma and Dadaab refugee camps [25]. The objective was to characterize the epidemiology of seasonal influenza and to detect novel influenza virus strains. In 2007, we expanded testing at the two refugee camps to include other respiratory viruses, with the aim of determining a broader range of viral aetiologies of acute respiratory infections (ARI). We present the results of 3 years of this surveillance.

## Methods

### Site and population

From September 2007 to August 2010, surveillance was conducted in Dadaab and Kakuma refugee camps, located in east and northwest Kenya, respectively. Dadaab is a large refugee camp complex and consists of three camps--Hagadera, Ifo and Dagahaley. Each camp has at least four health posts, which serve only outpatients, and one hospital with both inpatient and outpatient facilities. The refugee population in Dadaab has grown by more than 150% since the onset of surveillance, and as of August 2010, there were approximately 305,000 registered refugees. Kakuma had about 70,000 refugees throughout the study period and has three outpatient clinics and one hospital. Health care facilities in both camps serve the refugee population and the local Kenyan population.

In each camp, one hospital and one outpatient clinic were chosen as sentinel sites for surveillance. In Dadaab refugee camp complex, Hagadera hospital and one Hagadera health post were chosen as sentinel sites for Dadaab because at the start of the project, they were the busiest health facilities. In Kakuma, in addition to the only hospital, one outpatient clinic was selected. The two hospitals are the only facilities that offer inpatient services for refugees in Kakuma and Hagadera, Dadaab. They account for almost all hospitalisations.

### Case definitions

At both sites we enrolled patients with influenza-like illness (ILI) and severe acute respiratory illness (SARI). Definitions for ILI and SARI were adapted from those of the WHO [26-28].

• ILI was defined as fever ≥ 38°C and cough or sore throat.

• For children > 1 week and < 2 months old, SARI was defined as an admission to the paediatric ward with any of the following: respiratory rate > 60 per minute, severe chest indrawing, nasal flaring, grunting, fever ≥ 38°C, hypothermia < 35.5°C, or pulse oxygenation < 90%.

• For children 2 months to < 5 years of age, SARI was defined as cough or difficulty breathing and any one of the following: respiratory rate > 50/min for infants 2 months to < 1 year old or > 40/min for children 1 to < 5 years old, chest indrawing or stridor in a calm child, unable to drink or breast feed, vomiting, convulsions, lethargic or unconscious, or pulse oxygen saturation < 90%.

• For older children and adults ≥ 5 years of age, SARI was defined as fever ≥ 38°C, and cough or sore throat, and shortness of breath or difficulty breathing.

hospitalisation was a required part of the SARI case definition in all ages. For every patient, surveillance officers recorded specific signs and symptoms so that case classification could be validated. The maximum number of eligible ILI patients was limited to the first three cases per day for both sites.

### Clinical specimens

Trained surveillance officers identified eligible patients, administered a standardised questionnaire, and obtained nasopharyngeal (NP) and oropharyngeal (OP) swab specimens for diagnostic testing. NP and OP swab specimens were obtained according to the following procedure: a) a sterile nylon flocked plastic-shafted OP swab (503CS01, Copan Diagnostics, Murrieta, CA, USA) was used to rub the back of the oropharyngeal mucosal membrane for 3-5 s and then placed into 1 mL of viral transport media; b) a polyester-tipped flexible aluminium-shafted NP swab was inserted into the nose until it reached the nasopharynx, where it was rotated 180° and left in place for 3-5 s. The swab was inserted into a 1-mL vial of viral transport media. The cryovials were stored in a refrigerator at the health care facility and kept at 2-8°C for up to 96 h; the vials were then shipped to the Kenya Medical Research Institute (KEMRI)/CDC laboratory in Nairobi. Transportation to and from Dadaab and Kakuma is severely restricted; air transport is not always guaranteed, and flights are regularly diverted or cancelled with little or no notice. Considering their time-sensitivity, specimens were obtained only if they could be shipped within 96 h of collection. For two of the 3 years reported in this study, the NP and OP specimens were inserted into the same vial and tested as one specimen while in the final year the specimens were placed in separate vials in which case a positive result from either specimen was sufficient to classify a case as positive [29].

### Specimen processing

Specimens were not tested if the following conditions existed when the specimen arrived at the lab: the swab was missing; the volume was less than 600 μL; the specimen was at room temperature; patient identification was absent or incomplete; or the patient questionnaire was absent. In addition, the test results were discarded for any specimen whose human ribonuclease P (RNP) gene (internal control) was negative. Testing for respiratory viruses was carried out by real-time reverse transcriptase polymerase chain reaction (RT-PCR) at the KEMRI/CDC laboratory. An aliquot of each respiratory specimen was tested for influenza A and B viruses, respiratory syncytial virus (RSV), adenovirus (AdV), human parainfluenza viruses 1, 2 and 3 (PIV), and human metapneumovirus (hMPV). Details of the procedures used are described elsewhere [29-31].

### Data collection, entry, and analysis

For every ILI and SARI case enrolled, a brief standardized questionnaire was completed by staff at the health care facility. Demographic information, medical information, and a history of influenza vaccination were obtained directly from adult patients and from the parent or guardian for minors. Data were entered into a Microsoft Access database (Microsoft Corporation, Washington, USA) and analysed by SAS system for Windows, version 9.1 (SAS Institute Inc, Cary, North Carolina, USA) and Microsoft Excel (Microsoft Corporation, Washington, USA). Crude estimates of rates of hospitalisation per 1000 due to SARI for children < 5 years old were calculated by dividing the number of SARI cases among children < 5 years old by the population of that age group in the camp. Because the two hospitals are the only inpatient facilities in the respective camps and account for almost all admissions, we did not adjust for out-of-camp hospitalisation. Monthly population data for Hagadera, Dadaab and Kakuma camps for the months of September 2007 to August 2010 were obtained from the UNHCR Health Information System [32]. Population figures for Hagadera for December 2008 were not available, so we estimated the December 2008 population as the average of the months of November 2008 and January 2009. The 95% confidence intervals were calculated by using the Poisson distribution [33,34]. Rates were also estimated for the < 1 year and 1- to < 5-year age groups and by location and viral aetiology. When calculating these rates, Kenyan nationals were not included in the numerator, because population estimates for the local (non-refugee) population were not known. We were interested in calculating rates for acute onset illnesses; therefore, SARI cases with symptom onset of > 14 days were excluded. Rates were not calculated for children ≥ 5 years old and adults because reliable denominator data was not available.

Ethical approval for the surveillance activities was obtained from the KEMRI Ethical Review Committee (SSC Protocol Number 1161). Institutional review was waived by CDC because the study was considered to be a non-research public health activity. Informed written consent was obtained from all participants and from the guardians of minors.

## Results

During the study period, 6,647 patients meeting case definitions for ILI and SARI were enrolled. Specimens from 268 participants were discarded in the field before shipment (shipment did not occur in time) and an additional 115 specimens were rejected by the laboratory because of poor sample quality (see methods). Specimens from 6,264 participants were tested: 3,323 (53.0%) were from Kakuma and 2,941 (47.0%) were from Dadaab; 3,394 (54.2%) were from males (Table 1). There were 2,292 (36.6%) children < 1 year, 2846 (45.4%) 1-4 years, and 1,126 (18.0%) ≥ 5 years old. The median age was 1 year (range: 1 month-84 years), and the median number of days between symptom onset and enrolment was 2 days (range: 0-38 days). A total of 6,179 (99.2%) and 6,217 (99.8%) were enrolled within 7 days and 14 days of symptom onset, respectively. Date of symptom onset was not recorded for 32 patients. Participants were mainly refugees from Somalia (3,891 [62.1%]), Sudan (1,441 [23.0%]), Ethiopia (183 [2.9%]), and from other countries in east and central Africa (158 [2.5%]). Only 591 (9.4%) were Kenyan nationals receiving care at the refugee health facilities (Table 1). No participant had been vaccinated for influenza prior to being enrolled and tested.

**Table 1:** Demographic distribution and viral prevalence in specimens tested, by camp, Kenya, September 2007-August 2010

	Total (N = 6264) *n (%)*	Location		Case classification	
		
		Kakuma (N = 3323) *n (%)*	Dadaab (N = 2941) *n (%)*	ILI^1 ^ (N = 1815) *n (%)*	SARI^2 ^ (N = 4449) *n (%)*
**Gender**					
**Male**	3394 (54.2)	1800 (54.2)	1594 (54.2)	939 (51.7)	2455 (55.2)
**Female**	2870 (45.8)	1523 (45.8)	1347 (45.8)	876 (48.3)	1994 (44.8)

**Age**					
**< 1 years**	2292 (36.6)	1228 (37.0)	1064 (36.2)	520 (28.7)	1772 (39.8)
**1 - < 2 years**	1379 (22.0)	798 (24.0)	581 (19.8)	361 (19.9)	1018 (22.9)
**2 - < 5 years**	1467 (23.4)	815 (24.5)	652 (22.2)	497 (27.4)	970 (21.8)
**≥ 5 years**	1126 (18.0)	482 (14.5)	644 (21.9)	437 (24.1)	689 (15.5)

**Nationality**					
**Somali**	3891 (62.1)	980 (29.5)	2911 (99.0)	996 (54.9)	2895 (65.1)
**Sudanese**	1441 (23.0)	1431 (43.1)	10 (0.3)	407 (22.4)	1034 (23.2)
**Kenyan**	591 (9.4)	578 (17.4)	13 (0.4)	315 (17.4)	276 (6.2)
**Ethiopian**	183 (2.9)	178 (5.4)	5 (0.2)	59 (3.6)	124 (2.8)
**Other^3^**	158 (2.5)	156 (4.7)	2 (0.1)	38 (2.1)	120 (2.7)

**Infection prevalence**					
**Adenovirus**	1361 (21.7)	815 (24.5)	546 (18.6)	352 (19.4)	1009 (22.7)
**RSV**	781 (12.5)	364 (11.0)	417 (14.2)	155 (8.5)	626 (14.1)
**hMPV**	359 (5.7)	180 (5.4)	179 (6.1)	93 (5.1)	266 (6.0)
**Parainfluenza**	591 (9.4)	319 (9.6)	272 (9.3)	155 (8.5)	436 (9.8)
**Influenza A**	607 (9.7)	300 (9.0)	307 (10.4)	197 (10.9)	410 (9.2)
**Influenza B**	161 (2.6)	75 (2.3)	86 (2.9)	55 (3.0)	106 (2.4)
**At least one virus**	3119 (49.8)	1642 (49.4)	1477 (50.2)	837 (46.1)	2282 (51.3)
					

^1^Influenza-like illness; ^2^Severe acute respiratory infection; ^3^Burundi, Democratic Republic of Congo, Rwanda, Uganda

**Figure 1:**
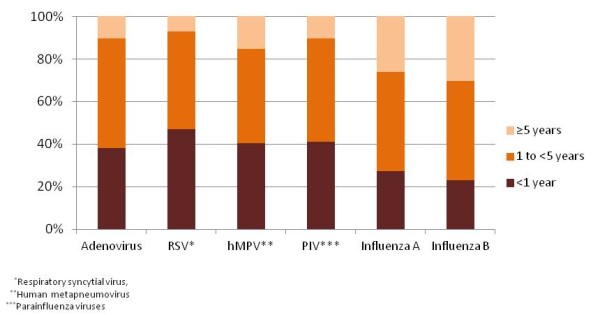
**Virus isolation by age group from persons with influenza-like illness or severe acute respiratory infection, Kakuma and Dadaab refugee camps, Kenya, 2007-2010**. Age group distribution was < 1 year, n = 2292 (36.6%); 1- < 5 years, n = 2846 (45.4%); ≥ 5 years, n = 1126 (18.0%).

Of the 6,264 tested, illnesses for 4,449 (71.0%) were classified as SARI and for 1,815 (29.0%) as ILI; 3,119 (49.8%) were positive for at least one virus (Table 1). The numbers and proportions of specimens positive for respiratory viruses were: AdV, 1361 (21.7%); RSV, 781 (12.5%); hMPV, 359(5.7%); PIV, 591 (9.4%); influenza A, 607 (9.7%); and influenza B, 161 (2.6%) (Table 1). Children < 1 year old accounted for 47.0% of RSV-positive patients, compared with 38.1% for AdV, 40.4% for hMPV, 41.1% for PIV, 27.3% for influenza A, and 23.0% influenza B (Figure 1). Table 1 below also shows proportion positive for virus by refugee camp and by case classification.

### Rates of hospitalisation

The annual crude rate of SARI hospitalisation for 2007-2010 was 57 per 1000 children < 5 years old per year (Table 2). Virus-positive crude hospitalisation rates (per 1,000 children < 5 years old) were 14 for AdV, 9 for RSV, 6 for PIV, 4 for hMPV, 5 for influenza A, and 1 for influenza B. The annual crude rate of SARI hospitalisation was highest in children < 1 year old (156 per 1000 children per year). Virus-positive hospitalisation crude rates per 1000 child-years for children < 1 year old were 35 for AdV, 29 for RSV, 17 for PIV, 11 for hMPV, 11 for influenza A, and 2 for influenza B. The ratio of rates for children < 1 year and 1 to < 5 years old was 3.7:1 for AdV, 5.5:1 for RSV, 4.4:1 for PIV, 5.1:1 for hMPV, 3.2:1 for influenza A, and 2.2:1 for influenza B. The average crude SARI hospitalisation rates for children < 5 years old were 1.6 times higher in Kakuma than in Dadaab (*p *< 0.001). The rates for AdV and PIV were 2.2 (*p *< 0.001) and 2.1 (*p *< 0.001) times higher for Kakuma than for Dadaab. The 2009 pandemic influenza A (pH1N1) was not associated with increased hospitalisation in either camp during the periods when pH1N1 was detected.

**Table 2:** Rates of SARI overall and by specific virus per 1000 children per year, Kakuma and Dadaab Refugee Camps, Kenya, 2007-2010

		AdV (95% CI^1^)	RSV(95% CI^1^)	**hMPV(95% CI**^**1**^)	PIV(95% CI^1^)	Flu A(95% CI^1^)	**Flu B (95% CI**^**1**^)	SARI(95% CI^1^)
**Kakuma**	**< 1 yr**	52.2 (41.7 - 65.4)	32.1 (24.1 - 42.8)	9.7 (5.7 - 16.3)	25.0 (18.1 - 34.7)	12.3 (7.7 - 19.5)	1.2 (0.3 - 5.3)	188.0 (167.0 - 211.7)
	**1 to 5 yrs**	13.8 (11.3 - 16.9)	6.1 (4.5 - 8.2)	3.1 (2.0 - 4.7)	5.4 (3.9 - 7.5)	4.2 (2.9 - 6.1)	1.4 (0.8 - 2.7)	49.8 (44.8 - 55.4)
	**< 5 yrs**	20.6 (17.7 - 23.9)	10.7 (8.6-13.1)	4.2 (3.0-5.9)	8.9 (7.0-11.1)	5.6 (4.2 - 7.5)	1.4 (0.8 - 2.5)	74.2 (68.5 - 80.3)

**Dadaab**	**< 1 yr**	23.5 (17.8 - 30.9)	26.8 (20.7 - 34.7)	11.4 (7.7 - 17.0)	11.3 (7.6 - 16.8)	10.3 (6.8 - 15.6)	2.9 (1.3 - 6.3)	134.3 (119.7 - 150.6)
	**1 to 5 yrs**	6.6 (5.2 - 8.3)	4.7 (3.6 - 6.2)	1.5 (0.9 - 2.4)	2.8 (2.0 - 4.1)	2.9 (2.1 - 4.2)	0.7 (0.3 - 1.4)	28.4 (25.4 - 31.8)
	**< 5 yrs**	9.5 (7.9 - 11.3)	8.5 (7.0 - 10.3)	3.2 (2.3 - 4.3)	4.3 (3.3 - 5.6)	4.2 (3.2 - 5.5)	1.1 (0.6 - 1.8)	46.5 (42.9 - 50.4)

**Overall**	**< 1 yr**	35.0 (29.4 - 41.7)	29.0 (23.9 - 35.1)	10.7 (7.8 - 14.7)	16.8 (13.1 - 21.6)	11.1 (8.2 - 15.1)	2.2 (1.1 - 4.4)	155.9 (143.5 - 169.3)
	**1 to 5 yrs**	9.4 (8.1 - 11.0)	5.2 (4.3 - 6.4)	2.1 (1.5 - 2.9)	3.9 (3.0 - 4.9)	3.4 (2.7 - 4.4)	1.0 (0.6 - 1.6)	36.8 (34.1 - 39.8)
	**< 5 yrs**	13.8 (12.3 - 15.5)	9.3 (8.1 - 10.8)	3.6 (2.9 - 4.5)	6.1 (5.1 - 7.3)	4.8 (3.9 - 5.8)	1.2 (0.8 - 1.8)	57.4 (54.3 - 60.8)

^**1**^95% confidence Interval

### Seasonal distribution

**Figure 2:**
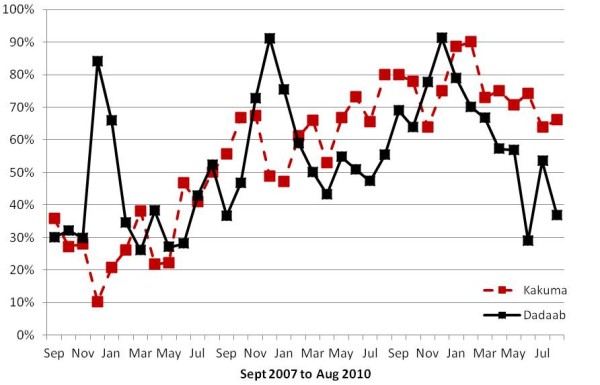
**Proportion of specimens positive for at least one virus among children < 5 years old with severe acute respiratory infection, Dadaab and Kakuma Refugee Camps, Kenya, September 2007 to August 2010**.

**Figure 3:**
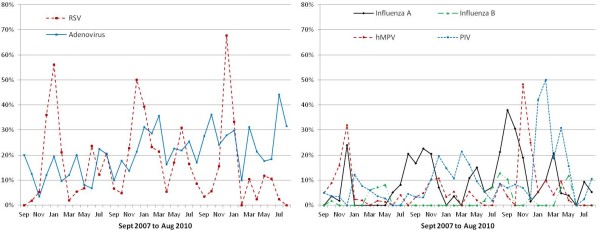
**Proportion of specimens positive for adenovirus, respiratory syncytial virus (RSV), parainfluenza viruses (PIV), human metapneuomovirus (hMPV), influenza A, and influenza B among children < 5 years old with severe acute respiratory infection in Dadaab refugee camp, Kenya, September 2007 to August 2010**.

In Dadaab, the rates for most viruses peaked from November to February (Figure 2). The rates of RSV-associated hospitalisation were highest during that time, with a minor increase in June. The rates for hMPV and influenza A peaked in November and December (Figure 3). PIV was mostly detected in January and February, and no distinct seasonal pattern was seen for AdV (Figure 3). In Kakuma, no distinct seasonality patterns were observed.

## Discussion and conclusions

Our study suggests that viruses are a major cause of significant respiratory infections in two large refugee camps in Kenya; at least one virus was detected in specimens from 50% of the participants. Although data from UNHCR's Health Information System suggest that ARI is the highest cause of morbidity and mortality in the refugee camps, this is the first study that we are aware of to demonstrate the viral aetiologies of these infections. AdV and RSV were the leading pathogens identified, accounting for approximately two-thirds of viruses detected. As expected, rates associated with viral infection were highest for children < 12 months of age [4,6,35,36]. The crude rates of hospitalisation per 1000 for SARI were more than four times higher for children < 1 compared to children 1 to < 5 years old.

Our findings for these two sites were similar to those reported from non-refugee populations in Mozambique, Gambia, Nigeria, South Africa, Indonesia, and India [37-39]. Rates per 1000 for severe infections due to RSV for children < 1 year and < 5 years of age were reported to be 15 and 9 (South Africa), 15 and 5 (Mozambique), and 16 and 10 (Indonesia) [39]. In a 3-year cohort study in rural India, severe lower respiratory tract infection (LRTI) rates per 1000 due to RSV for children aged < 1, 1-2 years, and 2-3 years were found to be 14, 7, and 0 respectively [37]. In a meta-analysis of incidence of RSV associated severe LRTI in developing countries, Nair et al. estimated incidence in the < 1 year age group per 1000 per year as 22 (95% CI 9-53) [40]. Rates of respiratory virus infection in children < 5-years-old in the two camps were ~2 times those reported for the United States or Europe but were similar to rates found in special populations groups within these countries [4,41,42]. For instance, in the United States, rates of hospitalisation for respiratory infections were found to be up to 5 times higher among Alaskan Native people than the US average [43].

The surveillance period overlapped with the 2009 pandemic influenza A (H1N1). While 2009 pH1N1 virus became the predominant circulating influenza A virus subtype, it was not associated with unusual rates in hospitalisation among the refugee population.

This study adds to our knowledge of the seasonal variations of respiratory viral infections in Africa. Even though both sites have very similar climatic conditions (arid and dry), we found remarkable differences in seasonal variation of ARI and specific virus activity, which might indicate varied transmission patterns in different subregions of the continent. There was distinct seasonality in Dadaab, with peak transmission occurring in November and December. Kakuma, on the other hand, despite having up to 60% more hospitalisation due to SARI, had no such seasonality. These differences may be due to the locations of the two camps. Kakuma, in the northwest of the country near the Ugandan and Sudanese borders, may reflect trends of viral transmission in the broader equatorial regions of central Africa, while Dadaab, located in the east near the Somali border, may reflect trends of transmission in the horn of Africa. Similar differences in seasonality between neighbouring tropical countries in Africa and Asia have been reported previously [39,44]. Additional surveillance points are required to further evaluate temporal variations of ARI due to viral illness.

Refugee populations are especially at risk for severe illness from respiratory infections [12,20,21]. Unique challenges make individuals residing in camp settings especially vulnerable to exposure to airborne diseases. The camp system of registration, food, water, and firewood distribution encourages crowding of large groups in small, confined spaces. In addition, malnutrition, high population density and poor shelter conditions may contribute to the elevated rates seen in this population. The availability of new, sensitive, reliable, and cost-effective technologies that can test multiple pathogens simultaneously from one easily obtainable specimen means that surveillance systems can now be set up for previously inaccessible populations [45].

Our study has several limitations: 1) Not all eligible individuals meeting the case definitions were identified, and not all those who were identified agreed to participate in the study. Thus the actual rates of SARI and virus infection associated SARI is likely higher than reported. 2) We did not attempt to collect data on all cases of ILI in the camp; therefore, we were not able to estimate rates of ILI. 3) Although the two hospitals are the only ones in the camps, refugees may have sought health care elsewhere; we did not capture information on health utilization among the refugees. If refugees did seek treatment elsewhere, our rates would again underestimate the actual rates. 4) The results of this study should also be interpreted with caution especially for pathogens e.g. AdV with known background carriage in healthy, asymptomatic individuals. Finally, the surveillance system was not designed to measure all indicators of ARI disease; due to lack of data, we could not estimate key outcomes, including mortality and duration of hospitalisation.

Our study found that viruses are a major cause of respiratory infection in the two refugee camps. To decrease the burden of respiratory illnesses requires a multipronged interventional plan. Physical interventions like handwashing with soap have been found to decrease the odds of respiratory infection by as much 55%, and nosocomial transmission has been shown to decrease by 66% when cohort nursing and wearing of gloves and gowns were introduced [46-48]. Measures could be put in place to minimise crowding, which is very common in refugee camps and has been associated with increased transmission of respiratory infections, and to target public health education messages during peak transmission months [49]. All SARI cases in this study were hospitalised in a ward shared by severely sick children presenting with other conditions. While we have not explored hospital acquired infection in this study, measures could also be taken to minimise potential nosocomial transmission. Because of the high rates of morbidity associated with these viruses in the refugee population, in addition to the implementation of innovative and effective approaches to achieve sustained compliance with hand hygiene promotions, refugees should be prioritised for vaccines when they become available [10,16-18] ARI prevention and control in refugee populations should be a key priority area for UNHCR, its partner agencies, and the international community.

## Competing interests

The authors declare that they have no competing interests.

## Authors' contributions

Conceived and designed the surveillance: RBE, RFB, MW, MAK, MKN, BKK, HB, MQ, BS, BW and DM. Established the surveillance programmes: JAA, MN, AM, MAK, EA, MKN, MW, BKK, HB, RN, MQ, BS, BW, DM, RFB, RBE. Performed and interpreted laboratory tests: MKN, LWM. Analysis and interpretation of data: JAA, AG, EA, RN, MAK, RBE, RFB. Drafted the initial manuscript: JAA, MAK, AG, RFB, RBE, MW, BKK, HB, RN, LWM, AM, BS. All authors contributed to and approved the final manuscript.

## Pre-publication history

The pre-publication history for this paper can be accessed here:

http://www.biomedcentral.com/1471-2334/12/7/prepub
